# Non-Isochronal Behavior of Charge Transport at Liquid–Liquid and Liquid–Glass Transition in Aprotic Ionic Liquids

**DOI:** 10.1021/acs.jpcb.4c00939

**Published:** 2024-05-14

**Authors:** S. Koymeth, B. Yao, M. Paluch, S. Dai, N. Mokhtarinori, M. Swadzba-Kwasny, Z. Wojnarowska

**Affiliations:** † Institute of Physics, University of Silesia in Katowice, Silesian Center for Education and Interdisciplinary Research, 75 Pułku Piechoty 1A, 41-500 Chorzów, Poland; ‡ Chemical Sciences Division, Oak Ridge National Laboratory, Oak Ridge, Tennessee 37831, United States; § Department of Chemistry, Institute for Advanced Materials & Manufacturing, University of Tennessee, Knoxville, Tennessee 37996, United States; ∥ The QUILL Research Centre, School of Chemistry and Chemical Engineering, The Queen’s University of Belfast, David Keir Building, Stranmillis Rd, BT9 5AG Belfast, NI, U.K.

## Abstract

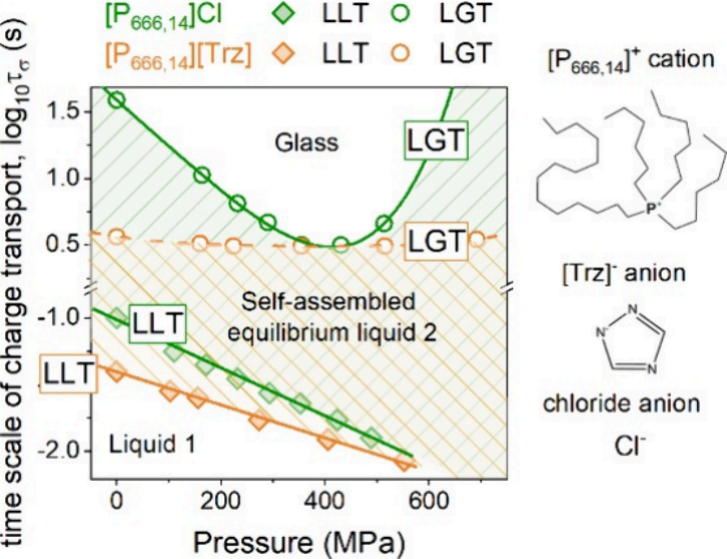

A reversible, first-order transition separating two liquid phases of a single-component material is a fascinating yet poorly understood phenomenon. Here, we investigate the liquid–liquid transition (LLT) ability of two tetraalkylphosphonium ionic liquids (ILs), [P_666,14_]Cl and [P_666,14_][1,2,4-triazolide], using differential scanning calorimetry and dielectric spectroscopy. The latter technique also allowed us to study the LLT at elevated pressure. We found that cooling below 205 K transforms [P_666,14_]Cl and [P_666,14_][Trz] from one liquid state (liquid 1) to another (the self-assembled liquid 2), while the latter facilitates the charge transport decoupled from structural dynamics. In contrast to temperature, pressure was found to play an essential role in the self-organization of a liquid 2 phase, resulting in different time scales of charge transport for rapidly and slowly compressed samples. Furthermore, *τ*_*σ*_(*P*_LL_) was found to be much shorter than *τ*_*σ*_(*T*_LL_, *P*=atm), which constitutes the first example of non-isochronal behavior of charge transport at LLT. In turn, dielectric studies through the liquid–glass transition revealed the non-monotonic behavior of *τ*_*σ*_ at elevated pressure for [P_666,14_]Cl, while for [P_666,14_][Trz] *τ*_*σ*_(*P*_g_) was almost constant. These results highlight the diversity of liquid–liquid transition features within the class of phosphonium ionic liquids.

## Introduction

The first-order liquid–liquid phase transition (LLT),^1^ separating two disordered phases of single-component material, is among the most elusive physical phenomena. Only four molecular fluids—water,^2,3^ triphenyl phosphite (TPP),^4,5^*n*-butanol,^6^ and d-mannitol^7^—were found to have two liquid states with locally different local structure, density, relaxation dynamics, and thermodynamic properties. However, even in these liquids, observation of a genuine liquid-to-liquid phase transition is challenging: pure water has shown an amorphous–amorphous transition,^8^ whereas a change from liquid 1 to a glassy phase of liquid 2 has been reported for TPP.^9^ This last transition has been interpreted as a cold crystallization by some authors.^10^ A true LLT, in which one liquid transforms into another and both can flow, was found only in supercooled Al_2_O_3_–Y_2_O_3_ and liquid phosphorus.^11,12^

Very recently, LLTs have been discovered in ionic liquids (ILs)^13−15^ defined as molten salts that melt below 100 °C. What is more, genuine LLTs were found not in one isolated ionic liquid but in an entire set of supercooled ILs based on a common trihexyl(tetradecyl)phosphonium cation, [P_666,14_]^+^, and several anions, which allowed for an in-depth study of structure-related properties governing LLT.^14^ From X-ray diffraction and FT-IR spectroscopic studies of trihexyl(tetradecyl)phosphonium borohydride, [P_666,14_][BH_4_], it has been concluded that enhanced ordering of long alkyl chains encourages LLT.^13^ At the same time, any shortening of phosphate substituents or an increase in the size of an anion suppresses the liquid–liquid transition^16,17^ due to the lack of domain-building material and sterical hindrance of anion, respectively.

The self-assembly of phosphate cations originating LLT was found when the temperature drops below 203 K. Above this temperature limit, [P_666,14_]-based ILs (e.g., trihexyl(tetradecyl)phosphonium dicyanamide [P_666,14_][DCA], trihexyl(tetradecyl)phosphonium tricyanomethanide [P_666,14_][TCM], or trihexyl(tetradecyl)phosphonium thiocyanate [P_666,14_][SCN]) exist in a supercooled liquid state with the charge transport being viscosity-controlled, as for typical aprotic ionic liquids. On the other hand, below the LLT in a nanostructured supercooled phase, their ion diffusion becomes much faster than structural dynamics.^18^ It has been shown that long alkyl chains of cations form a skeleton that contributes substantially to structural dynamics, while anions travel easily through created channels and thus govern the charge transport. Such a decoupling between the time scale of charge transport (τ_σ_) and structural relaxation (τ_α_) is maintained down to the liquid–glass transition (*T*_g_). Consequently, at *T*_g_, *τ*_*α*_ achieves the time scale of 100 or 1000 s which is typical for ionic glass-formers,^19^ while τ_σ_ is a few decades shorter than this value. At the same time, *τ*_*σ*_ and τ_α_ are in the range 0.3–15 ms at *T*_LL_ for most of the tetraalkyl phosphonium ILs studied so far.

In addition to isobaric cooling, isothermal compression is another available route to induce LLT in [P_666,14_] ILs. Furthermore, in contrast to phosphorus or nitrogen, extreme conditions (1050 °C and 1 GPa for phosphorus^20^ and 1920 K and 50 GPa for nitrogen^21^) are not necessary for LLT to occur in ILs. Specifically, isothermal compression up to 600 MPa at 233 K is enough to induce LLT in [P_666,14_] ILs, and even 150 MPa is sufficient if the temperature is lower, i.e., 213 K.^18^ An exciting observation coming from high-pressure conductivity studies of phosphonium ILs is that, in every single case examined so far, the sign of LLT is observed at *τ*_*σ*_ = const. within a given system; i.e., it occurs at a certain time scale of ionic motions independently of *T*–*P* conditions.^14^ However, when the conductivity relaxation time at the liquid–glass transition pressure (*P*_g_) was considered, two different scenarios were found, depending on the anion size. For the IL with small [SCN]^−^ anions, high pressure facilitated their motions (due to stronger van der Waals forces between the cation alkyl chains under pressure and consequently more channels available for transport), which was reflected by the shortening of *τ*_*α*_(*P*_g_) compared to *τ*_*α*_(*T*_g_, *P*_atm_). However, above *P*_g_ = 170 MPa, the opposite effect was observed due to the reduced free volume available for anion motions. As a result, the decoupling between the time scales of conductivity and structural relaxation revealed a maximum at *P*_g_ = 170 MPa for [P_666,14_][SCN].^18^ On the other hand, as the anion size increases, the formation of channels for charge diffusion was found to be more challenging. Consequently, the effect of pressure on molecular packing of [P_666,14_][TCM] was weak, leading to the constant value of *τ*_*σ*_(*T*_g_, *P*_g_) under various temperature and pressure conditions. In this context, one can assume that the smaller the anion, the more significant the decoupling between *τ*_*σ*_ and *τ*_*α*_ should be observed, especially under high-pressure conditions. Furthermore, a clear minimum of *τ*_*σ*_(*P*_g_) is expected. Following this hypothesis, herein we examine the liquid–liquid and liquid–glass transition in two aprotic ionic liquids containing trihexyl(tetradecyl)phosphonium cation [P_666,14_]^+^ and two anions: small chloride Cl^–^ (van der Waals volume of 28 Å) and two times larger 1,2,4-trazolide [Trz] (van der Waals volume of 62 Å) over a wide temperature and pressure range. The dielectric experiments performed for [P_666,14_]Cl and [P_666,14_][Trz] at ambient pressure revealed that temperature and the time scale of charge transport at LLT are similar for both examined systems. However, the latter was much longer compared to those of other [P_666,14_]-based ILs. On the other hand, high-pressure measurements revealed exceptional behavior of charge transport at liquid–liquid transition pressure (*P*_LL_). Furthermore, evident non-monotonic behavior of the decoupling index has been observed for [P_666,14_]Cl, while *τ*_*σ*_(*T*_g_, *P*_g_) was almost constant for [P_666,14_][Trz]. Finally, the effect of the compression rate on the self-organization of the liquid 2 state was examined.

## Materials and Methods

### Synthesis of [P_666,14_][1,2,4-Triazolide]

The synthetic pathway we introduce involves the metathesis of two salts: an alkali salt of the targeted anion (sodium triazole) and a halide salt of the targeted cation ([P_66614_]Cl). The precursor trihexyl(tetradecyl)phosphonium chloride, min 95% CYPHOS IL101, was purchased from Strem Chemicals. Subsequently, the desired ILs can be obtained by evaporating the organic solvent. The stepwise process proceeds as follows: A round-bottomed flask was charged with NaH (10 mmol), flushed with N_2_, and then suspended in THF (5 mL). A solution of 1,2,4-triazole (10 mmol) in THF (5 mL) was added dropwise over 30 min and then stirred until the evolution of H_2_ ceased. A solution of trihexyl(tetradecyl)phosphonium chloride (10 mmol) in THF (10 mL) was added. The resulting mixture was stirred for 12 h and then quenched with deionized water (40 mL) and extracted with ethyl acetate three times (40 mL each). The remaining solvent was then removed by vacuum, and the resulting product was further dried under vacuum stirring at 60 °C for 24 h to provide an orange liquid. Nuclear-magnetic resonance (NMR) was used to characterize synthesized ionic liquids (Supporting Information).

### Differential Scanning Calorimetry

Calorimetric experiments of the studied ILs were performed by means of a Mettler Toledo DSC1STAR System equipped with a liquid nitrogen cooling accessory and an HSS8 ceramic sensor (a heat flux sensor with 120 thermocouples). During the experiments, the flow of nitrogen was kept at 60 mL min^–1^. Enthalpy and temperature calibrations were performed by using indium and zinc standards. The baseline was constructed as a straight line from the onset to the end point. A dedicated software, Mettler Toledo DSC1STAR, allows various calculations (onset, heat, peak temperature, etc.) from the original recorded DSC curves. Before the measurement, the samples were annealed 30 min at 373 K. Temperature ramps involved cooling to 185 K and heating to 373 K with a rate of 1, 2, 5, and 10 K/min. Samples were cycled at least 3 times to ensure reproducibility and high accuracy. The 6 h aging experiment was performed at 183 K after cooling with the rate of 10 K·min^–1^.

### Broadband Dielectric Spectroscopy

The dielectric measurements at ambient pressure for the studied ILs were carried out over a frequency range from 10^–2^ to 10^7^ Hz by means of a Novo-Control GMBH Alpha dielectric spectrometer. The Novocool system controlled the temperature with an accuracy of 0.1 K. During this measurement, the sample was placed between two stainless steel electrodes (diameter = 15 mm). The quartz ring provided the distance between plates. The measurements were performed on cooling and quenched heating. For the pressure-dependent dielectric measurements, we used the capacitor filled with the studied sample, which was next placed in the high-pressure chamber and compressed using silicone oil. Note that the sample was only in contact with stainless steel during the measurement. The Unipress setup measured the pressure with a resolution of 1 MPa. The temperature was controlled within 0.1 K by means of a Weiss fridge. The measurements were performed in two protocols: (i) on slow isothermal compression and (ii) on slow decompression preceded by fast compression to the glassy state.

## Results and Discussion

**Figure 1:**
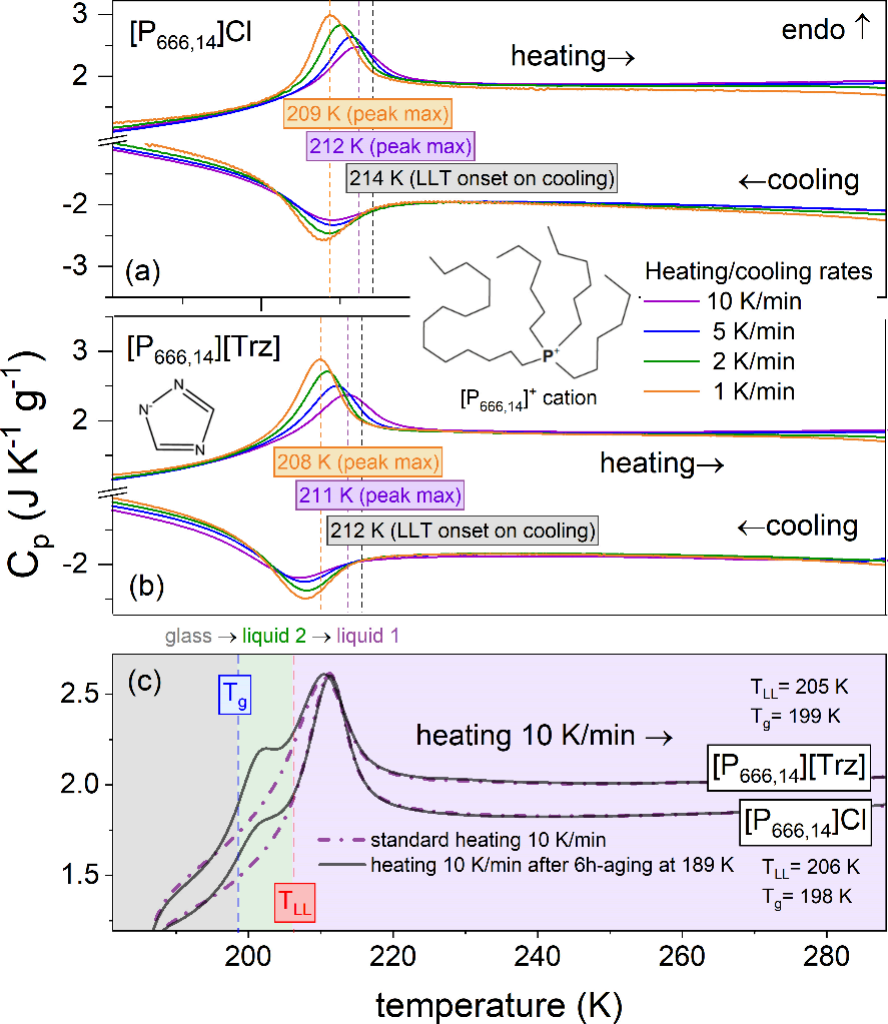
Differential scanning calorimetry (DSC) traces of [P_666,14_]Cl (a) and [P_666,14_][Trz] (b). Dashed lines indicate the onset of LLT determined on cooling (gray line) and the peak position obtained on heating (orange and violet lines). The chemical structure of [P_666,14_]^+^ cation and [Trz]^−^ anion is presented in the inset. Panel (c) presents DSC traces obtained during the standard heating with a rate of 10 K min^–1^ (dashed violet curved) and after the aging process performed in the glassy state, i.e., at 183 K (gray straight line). The blue dashed line indicates the liquid–glass transition temperature, while the red dashed line denotes the onset of LLT.

First, differential scanning calorimetry (DSC) was employed to check whether the chosen ILs undergo LLT as previously observed for other [P_666,14_]^+^-based compounds. The DSC scans performed on cooling and subsequent heating are presented in Figure 1a and b for [P_666,14_]Cl and [P_666,14_][Trz], respectively. As can be seen, independently of the applied cooling rate, a broad exotherm appears at the onsets of 214 and 212 K for [P_666,14_]Cl and [P_666,14_][Trz], respectively. However, as the cooling rate decreases, the peak’s enthalpy Δ*H* becomes larger. On heating, all thermal curves featured an endotherm, quite symmetrical with respect to the cooling cycles, with the peak maximum shifted toward higher temperatures for faster heating rates (209 → 212 K for Cl^–^; 208 → 211 K for [Trz]^−^). Upon further heating, no additional thermal effects were observed. These results indicate that neither of the examined ILs reveals the crystallization tendency, and the liquid–liquid transition is the only thermal effect seen for these samples during the standard cooling/heating scans. Interestingly, *T*_LL_ is similar for [P_666,14_]Cl and [P_666,14_][Trz] as well as for other [P_666,14_]-based systems, indicating that the transition temperature is independent of the anion type.

From Figure 1a and b, it is also apparent that *T*_g_ does not appear on the standard DSC thermograms of [P_666,14_][Trz] and [P_666,14_]Cl. Therefore, an aging experiment was performed to determine the *T*_g_ values of [P_666,14_]^+^-based ILs. It involves three stages: (i) cooling from RT to 183 K (expected to fall in the glassy state) with the rate of 10 K/min, (ii) time-dependent (6 h) isothermal annealing at 183 K, and (iii) subsequent heating with 10 K/min. Figure 1c compares the thermogram corresponding to the final stage of the aging experiment with the data obtained in a standard DSC scan. As a result of the physical aging, on the left side of the LLT peak, the step-like change of heat capacity emerged for both samples. Since the glassy aging is accompanied by an increase of heat capacity in the glass transition region (so-called overshoot peak), the newly formed thermal effect can be identified with the liquid–glass transition. The determined *T*_g_ value is equal to 198.5 ± 0.5 K for [P_666,14_][Trz] and [P_666,14_]Cl. Consequently, heating in the range 183–298 K transforms amorphous phase 2 into supercooled liquid 2 (above *T*_g_) and subsequently into the supercooled phase 1, while the latter is available above *T*_LL_. All three regions are marked in Figure 1c.

**Figure 2:**
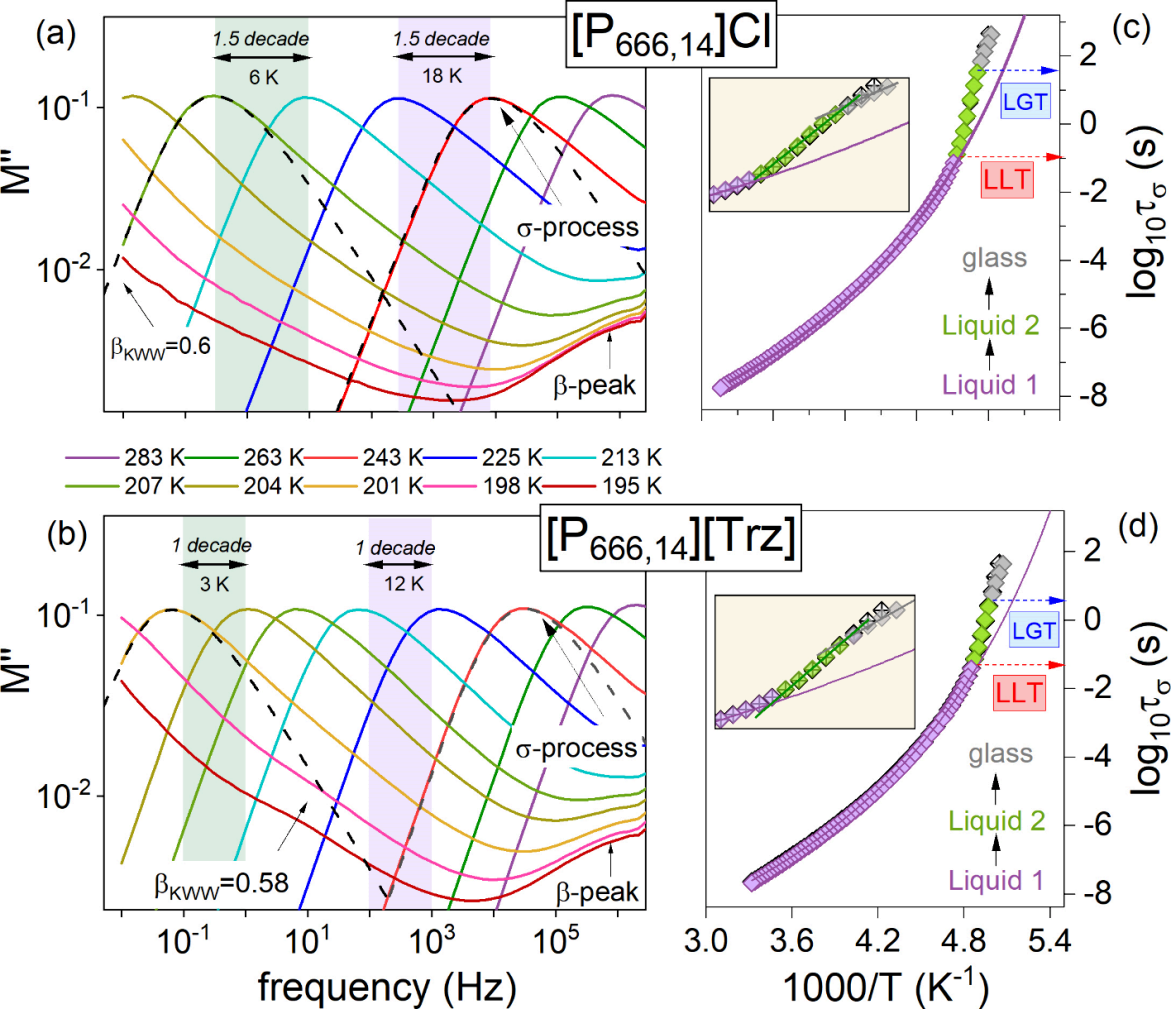
Representative dielectric data of [P_666,14_]Cl (a) and [P_666,14_][Trz] (b) recorded on cooling and presented in an electric modulus representation. Dashed lines present the fits of the KWW function ϕ(*t*) = exp[−(*t*/τ_α_)^*β*_KWW_^)] to experimental data with β_KWW_ exponents of 0.60 and 0.58 for [P_666,14_]Cl (a) and [P_666,14_][Trz] (b), respectively. Panels (c) and (d) present the temperature dependence of *τ*_*σ*_ for chloride and [Trz] salts of [P_666,14_]^+^-based ILs. Solid lines indicate the fits of the VFT function to experimental data recorded in the liquid 1 state. Red arrows denote the *τ*_*σ*_ at LLT, while blue arrows denote the *τ*_*σ*_ at *T*_g_. In the inset zoomed-in, the difference between τ_σ_(*T*^–1^) obtained on cooling (closed symbols) and heating (open symbols) is highlighted.

In the next part of our studies, dielectric measurements were performed to examine the ion dynamics across the liquid–liquid and liquid–glass transitions. For this purpose, two experimental protocols have been employed. The first one involves recording the dielectric response of the samples on cooling from 303 K (Δ*T* = 2 K in liquid 1 state and Δ*T* = 1 K in liquid 2 state) down to the glassy state, while, in the second procedure, ionic liquids were initially quenched to the glassy state and then frequency scans (10^–2^–10^6^ Hz) were performed upon heating. For ionic systems, complex electric conductivity σ*(*f*) = *ε*_0_(Z*(*f*)*C*_0_)^−1^ and complex electric modulus M*(*f*) = ε*(*f*)^−1^ are conventionally adopted to express the dielectric response.^22,23^ Since the latter formalism provides more detailed characteristics of supercooled and glassy states, it has been employed to evaluate the data collected for [P_666,14_]Cl and [P_666,14_][Trz]. Ten electric modulus spectra collected at various temperatures for each examined IL are selected in Figure 2a and b. As can be seen, the imaginary part of the complex modulus *M*″(*f*), frequently denoted as the σ-peak, shifts to lower frequencies upon cooling, which is typical for ion-containing systems and reflects the slowing down of ion diffusion.^24^ When *M*″(*f*) moves out from an experimental window, the system reaches a glassy state and the mobility of ions becomes strongly suppressed. Then, a secondary β-relaxation appears in the high-frequency part of the modulus curves. Interestingly, from first sight, in Figure 2a and b, it is difficult to recognize which supercooled phase, 1 or 2, represents the individual *M*″(*f*) peak. In contrast to previously examined [P_666,14_] ILs, the *M″*(*f*) spectra keep the same shape at various temperatures above and below the calorimetric *T*_LL_^DSC^. Furthermore, the amplitude of the *M*″(*f*) function does not change at all through the LLT. The only signature of LL transformation is an increased sensitivity of the *M*″ peak to temperature changes. For example, to shift the dielectric spectrum of [P_666,14_]Cl by 1.5 decades within the liquid 1 state, one needs to decrease the temperature by 18 K. However, only 6 K is enough to slow the ion dynamics by 1.5 decades in the liquid 2 state (see Figure 2a). To describe the ion dynamics of [P_666,14_]Cl and [P_666,14_][Trz] more precisely, we have used the frequencies corresponding to modulus peak maxima (*f*_max_) and determined the temperature evolution of conductivity relaxation times *τ*_*σ*_ = ^1^/_2_π*f*_max_ in their liquid 1 and liquid 2 states. To extract τ_σ_, in the vicinity of the liquid–glass transition, the σ-peak recorded in the liquid 2 state was shifted horizontally to cover the high-frequency flank of the spectra measured at *T* < *T*_g_. Such a data extrapolation could be employed since the time–temperature superposition (TTS) rule is satisfied in supercooled states of examined ILs. The resulting temperature dependence of *τ*_*σ*_ covering both supercooled states and the glassy phase is presented in Figure 2c and d for [P_666,14_]Cl and [P_666,14_][Trz], respectively. It can be seen that the ion dynamics of both examined salts reveals the Vogel–Fulcher–Tammann (VFT) type behavior at high temperatures, i.e., in the supercooled liquid 1. However, below the *T*_LL_^DSC^, the experimental points slow more than expected from the VFT fit. Consequently, the LLT in ILs can be considered a strong to fragile transition. Further cooling results in the vitrification of supercooled liquid 2, which is visible as a change in the slope of τ_σ_(*T*^–1^) dependence arround *T*_g_^DSC^. Interestingly, the transition between supercooled liquid 2 and its amorphous counterpart is better pronounced when the dielectric data of quenched ILs are analyzed (see insets of Figure 2c and d). Note that the change in τ_σ_(*T*^–1^) behavior visible at the *T*_g_ of the examined ILs is an inherent part of the liquid–glass transition and reflects substantial slowing down of charge transport in a glassy state.^25^

From analysis of *τ*_*σ*_(*T*^–1^) dependences presented in Figure 2c and d, we found that *τ*_*σ*_ at *T*_LL_ is almost the same for both examined ILs (log *τ*_*σ*_ = −1.25 and log τ_σ_ = −1 for [P_666,14_]Cl and [P_666,14_][Trz], respectively) and it is almost two decades slower compared to *τ*_*σ*_(*T*_LL_) determined for [SCN]^−^, [DCA]^−^, and [TCM]^−^ salts. The same trend can be observed when *τ*_*σ*_(*T*_g_) is considered. Specifically, log *τ*_*σ*_(*T*_g_) = 1.5, 0.5, and −0.36 for [P_666,14_]Cl, [P_666,14_][Trz], and [P_666,14_][DCA], respectively. Thus, for chloride and [Trz]^−^ salts, the charge transport at *T*_g_ is only slightly shorter than 100 s (log *τ*_*σ*_ = 2)—the time scale commonly identified with the freezing of ion mobility at *T*_g_. In this context, examining the transition from liquid 1 to liquid 2 phase and subsequently to amorphous phase 2 by isothermal compression would be interesting.

**Figure 3:**
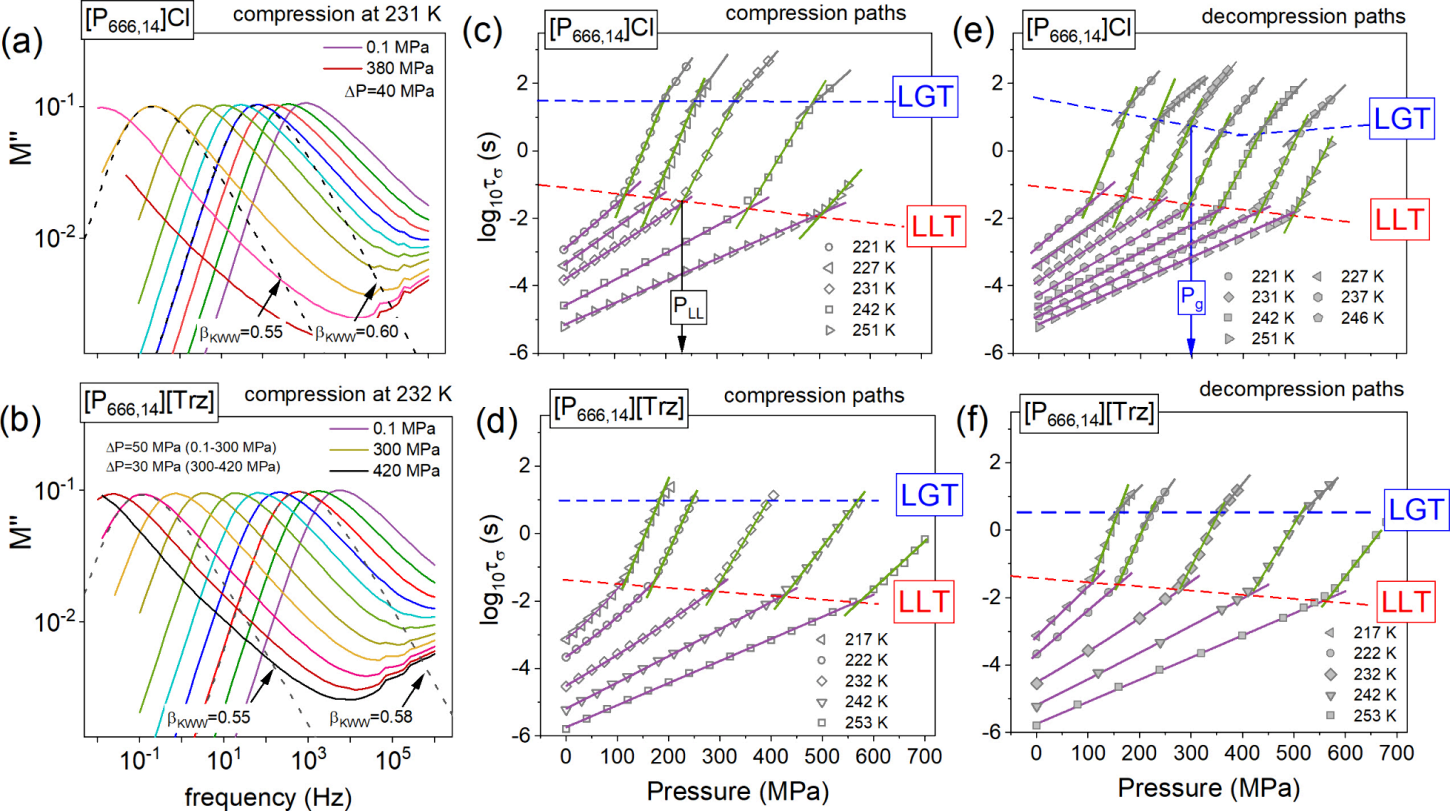
Representative dielectric data recorded for [P_666,14_]Cl (a) and [P_666,14_][Trz] (b) during isothermal compression at 231 and 232 K and presented in electric modulus representation. Dashed lines represent the fits of the KWW function converted to the frequency domain. Panels (c) and (d) present the pressure dependence of the conductivity relaxation times *τ*_*σ*_ measured during the slow compression under different isothermal conditions for [P_666,14_]Cl and [P_666,14_][Trz]. Panels (e) and (f) present the isothermal *τ*_*σ*_(*P*) dependences recorded during the decompression of rapidly squeezed samples.

Although temperature change is probably the most straightforward method for inducing a first-order LL phase transition, recently, it has been shown that isothermal compression is equally efficient. Furthermore, an important advantage of isothermal squeezing is that it changes only molecular packing, while the thermal energy of molecules remains constant. To fully reproduce LLT studies performed under ambient conditions (cooling vs quench heating), two different high-pressure experiments have been performed. Precisely, after the temperature stabilization at *T* > *T*_LLT_, ILs were slowly compressed through both supercooled states. Later on, the pressure was released to the starting point. Afterward, rapid compression directly to the glassy state was performed, and the dielectric spectra were collected along the decompression path. The representative spectra recorded during the compression of [P_666,14_]Cl and [P_666,14_][Trz] at 231 and 232 K are shown in Figure 3a and b. Analogous to isobaric cooling, isothermal squeezing shifts the *M*″(*f*) curve toward higher frequencies. However, in contrast to ambient pressure measurements, the modulus loss peak becomes broader above the liquid–liquid transition pressure (*P*_LL_). The substantial broadening of *M*″(*f*) spectra is mainly observed for chloride salt, for which the β_KWW_ exponent is decreasing from 0.60 in phase 1 to 0.55 in the supercooled liquid 2 at 231 K. Further analysis of the dielectric data shows their stronger pressure sensitivity in the liquid 2 state. To investigate this effect thoroughly, the pressure dependence of conductivity relaxation times was determined along each isotherm. As can be seen in Figure 3c,d, every single *τ*_*σ*_*–P* dependence obtained on the compression path of [P_666,14_]Cl and [P_666,14_][Trz] reveals a well-defined kink being a manifestation of LLT. However, it appears at different log *τ*_*σ*_ for each isotherm. Specifically, *τ*_*σ*_ at the LLT continuously decreases with *T*–*P* conditions. Interestingly, this change is almost the same for both examined salts (∼1 decade) and does not depend on the experimental protocol (compression vs decompression; see Figure 3e,f). Consequently, the character of LLT in [P_666,14_]Cl and [P_666,14_][Trz] is non-isochronal, which is an exception among [P_666,14_]-based ionic liquids.

**Figure 4:**
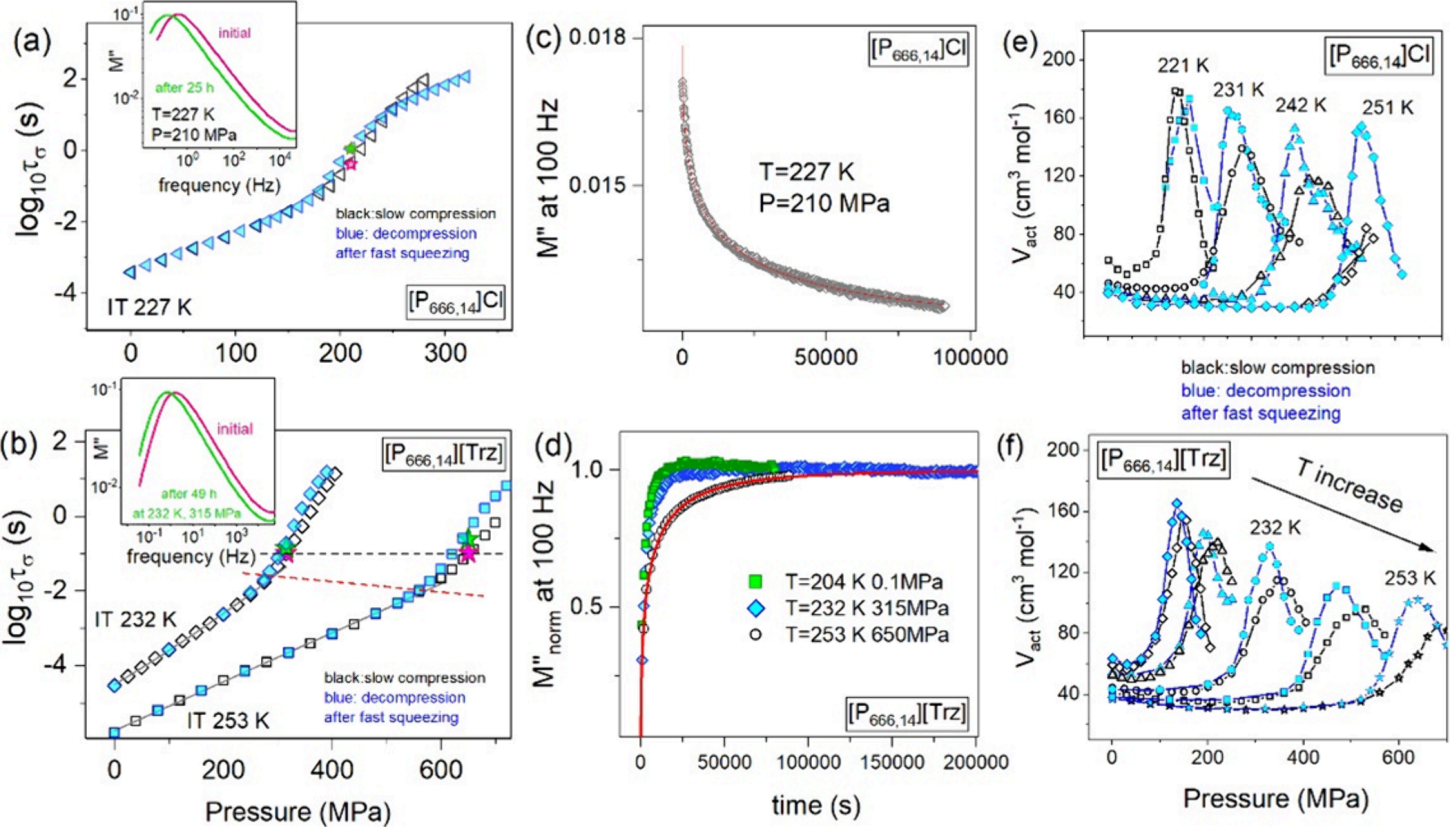
Panels (a) and (b) present the comparison between the pressure dependence of the conductivity relaxation times *τ*_*σ*_ measured during the slow compression (open symbols) and decompression preceded by fast squeezing (closed blue symbols). Red stars represent the position of the *M*″(*f*) peak immediately after the compression from 0.1 MPa. Green stars represent the *M*″(*f*) position once the equilibrium is achieved. Insets present the evolution of *M*″(*f*) spectra at isothermal time-dependent dielectric measurements. Panels (c) and (d) present the time evolution of *M*″ at 100 MHz in the liquid 2 state. The kinetic curves of [P_666,14_][Trz] were normalized using the  formula. Panels (e) and (f) depict the apparent activation volume *V*_act_ as a function of pressure for [P_666,14_]Cl and [P_666,14_][Trz], respectively.

When supercooled liquid 2 starts to freeze, isothermal *τ*_*σ*_*–P* dependences reveal the second kink being a manifestation of the liquid-to-glass transition of [P_666,14_]Cl and [P_666,14_][Trz]. However, in contrast to LLT, the kink of the *τ*_*σ*_–*P* curves observed at *P*_g_ being induced by compression occurs at constant conductivity relaxation times for a given system, specifically, at log *τ*_*σ*_ = 1.5 and log *τ*_*σ*_ = 1 for [P_666,14_]Cl and [P_666,14_][Trz], respectively. Surprisingly, when the ILs were rapidly squeezed and the dielectric data were obtained along the decompression path (Figure 3e,f), *τ*_*σ*_(*P*_g_) for [P_666,14_][Trz] is again constant while a clear minimum in *τ*_*σ*_(*P*_g_) dependence at around 360 MPa is observed for [P_666,14_]Cl. Herein, it should be noted that, for every single isotherm presented in Figure 3c–f, τ_σ_ at *P*_g_ is faster than 1000 s—the time scale commonly identified with the freezing of charge transport for aprotic ionic liquids. Since structural relaxation time, τ_α_, is in the order of 1000 s at the liquid–glass transition temperature and pressure for all glass-forming liquids (both ionic and nonionic),^26^ one can conclude that the examined phosphonium ILs are characterized by the charge transport decoupled from structural relaxation under both ambient and elevated conditions. Furthermore, the degree of decoupling depends on the compression rate. Precisely, when liquid 2 of [P_666,14_]Cl and [P_666,14_][Trz] is compressed slowly, it enters a glassy state at τ_σ_ shorter than it is for rapidly squeezed material. Consequently, two different glassy states differing in the time scale of charge transport are obtained within a single-component material. This phenomenon, called polyamorphism, gives a unique possibility of tuning the properties of amorphous electrolytes. In this context, the following question arises: Are the liquid 2 states obtained by different compression rates the same or different in terms of the time scale of charge transport? To address this issue, we have directly compared the *τ*_*σ*_(*P*) dependences measured on slow compression and during the decompression of rapidly squeezed material. As can be seen in Figure 4a,b in supercooled liquid 1 (below *P*_LL_), the *τ*_*σ*_–*P* curves obtained on various pressure pathways are practically identical, implying that phase 1 is an equilibrium liquid insensitive to the sample history. On the other hand, above *P*_LLT_, two different time scales of τ_σ_ are obtained, while the conductivity relaxation determined for slowly compressed material is faster than that of rapidly squeezed liquid. In addition, this difference seems to be more pronounced at higher temperatures and pressures. To quantify this effect, the apparent activation volume *V*_act_ = 2.303*RT*(d log *τ*_*σ*_/d*P*)_*T*_, reflecting the pressure sensitivity of ion dynamics, has been determined. As can be seen in Figure 4e,f, around 4-fold increase in *V*_act_ occurs at LLT, indicating the formation of extensive self-assembled structures much more sensitive to pressure changes when compared to the normal liquid 1 state. In turn, when the liquid 2 state is considered, higher values of *V*_act_ are obtained for the rapidly compressed material, demonstrating its greater sensitivity to pressure changes. This suggests that fast squeezing facilitates the formation of a self-assembled liquid state with significant free volume available for anion motions and explains an extensive decoupling between charge transport and structural dynamics obtained for this material (more prominent when compared to a slowly compressed sample). Since the difference in *τ*_*σ*_ and *V*_act_ between the fast and slowly compressed liquid 2 state is more and more pronounced as temperature and pressure increase, it becomes understandable that the same τ_σ_(*T*) dependences were obtained in liquid 2 state under ambient conditions for quenched and slowly cooled IL.

From the experiments described above, one can conclude that pressure plays an essential role in the self-organization of a liquid 2 phase, thus determining the degree of the decoupling phenomenon at *P*_g_ and below. Therefore, it is crucial to recognize which structure, obtained by fast or slow compression, is an equilibrium one. To address this issue [P_666,14_]Cl was slowly compressed at 227 K from ambient pressure to *P* > *P*_LL_, and then time-dependent dielectric scans were performed. For [P_666,14_][Trz], the equilibration experiment was performed twice, at 232 and 253 K. As presented in the inset to Figure 4a and b, in every examined case, the modulus spectrum moves toward lower frequencies in time. Consequently, the conductivity relaxation time elongates. The initial and final position of the *M*″(*f*) spectrum has been denoted as red and green stars in relaxation maps in Figure 4a and b. On the other hand, the time evolution of *M*″ determined at a single frequency (100 Hz) is depicted in Figure 4c for [P_666,14_]Cl and Figure 4d for [P_666,14_][Trz]; however, in the latter case, the data have been normalized to compare the kinetic curves recorded at various *T–P* conditions directly. It can be seen that the equilibration process depends strongly on thermodynamic conditions and may take up to a few days. It lasts the shortest at ambient pressure (3 h at 204 K for [P_666,14_][Trz]) and the longest at elevated pressure (48 h at 253 K and 650 MPa for [P_666,14_][Trz]). When the equilibration process is completed, the *M*″ peak achieves the frequency position corresponding to τ_σ_ of the rapidly squeezed sample (see Figure 4a and b). This indicates that the data recorded on decompression reflect the dynamics of the equilibrated liquid 2 state. Consequently, to determine the proper *T*_g_(*P*_g_) and τ_σ_(*P*_g_) lines, the τ_σ_(*P*) dependences obtained on decompression need to be evaluated.

**Figure 5:**
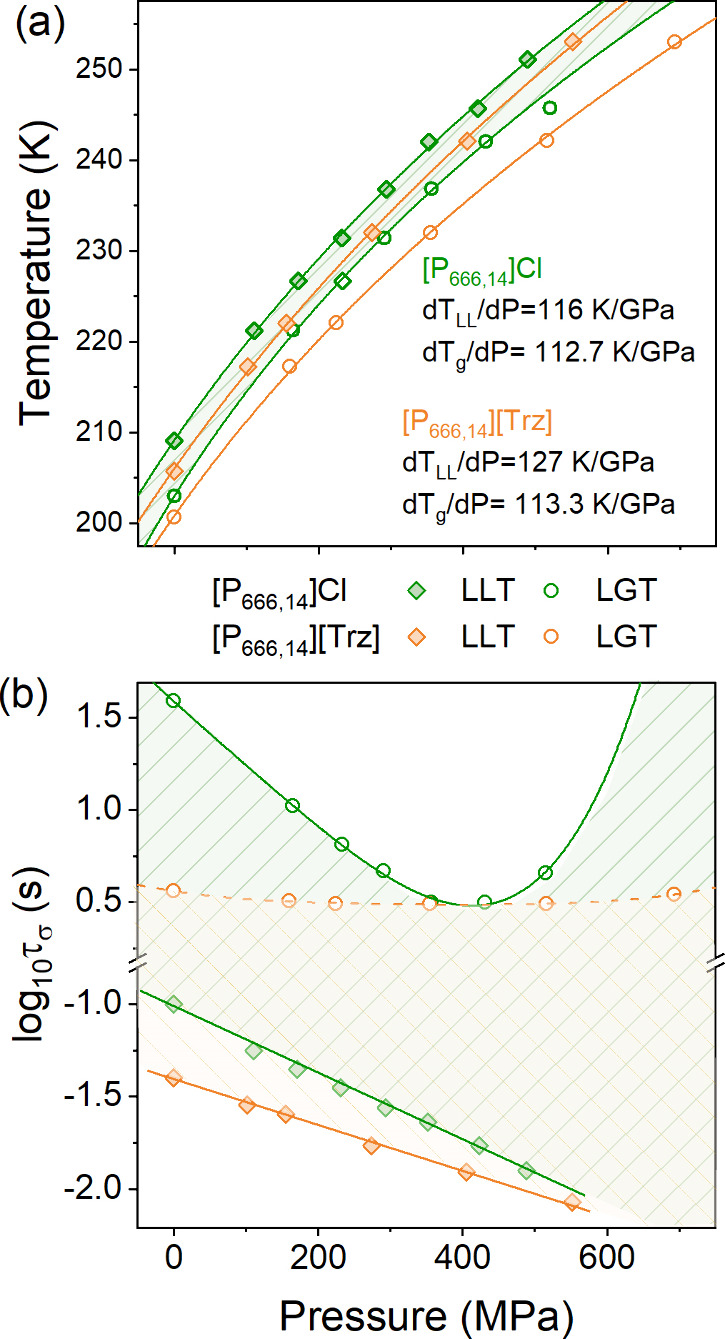
(a) *T*_LL_ and *T*_g_ as a function of *P*. Panel (b) presents log τ_σ_(*T*_g_, *P*_g_) and log τ_σ_(*T*_LL_, *P*_LL_) for [P_666,14_]Cl and [P_666,14_][Trz]. The green area denotes the liquid 2 phase of [P_666,14_]Cl, while the orange area denotes the liquid 2 phase of [P_666,14_][Trz].

Defining *P*_LL_ and *P*_g_ as the pressure at which the log *τ*_*σ*_ rapidly changes the behavior, the *T*_LL_(*P*_LL_) and *T*_g_(*P*_g_) dependences were obtained for [P_666,14_]Cl and [P_666,14_][Trz] and are presented in Figure 5a. Additionally, log *τ*_*σ*_(*P*_LL_) and log *τ*_*σ*_(*P*_g_) were determined for both ILs and are depicted in Figure 5b. Due to the nonlinear character of *T*_g_(*P*_g_) and *T*_LL_(*P*_LL_) lines, the empirical Andersson–Andersson equation,^27^*T*_g_ = *k*_1_(1 + (*k*_2_/*k*_3_)*P*)^1/*k*_2_^, was employed to parametrize the data and the *k*_1_/*k*_3_ ratio was used to calculate the d*T*_g_/d*P* and d*T*_LL_/d*P* coefficients. As can be seen in Figure 5a, the values of d*T*_g_/d*P* determined in the limit of ambient pressure are almost the same for [P_666,14_]Cl and [P_666,14_][Trz] (113 K/GPa), and they are close to that determined previously for [P_666,14_][DCA] (112 K/GPa), [P_666,14_][TCM] (118 K/GPa), and other aprotic ionic liquids.^28,29^ On the other hand, the transformation of liquid 1 to liquid 2 state reveals different characteristics when compared to other [P_666,14_] ILs. Precisely, d*T*_LL_/d*P* coefficients are equal to 116 and 127 K/GPa for [P_666,14_]Cl and [P_666,14_][Trz], respectively, which is higher than the d*T*_LL_/d*P* of any other [P_666,14_] IL examined so far. The determined values of d*T*_LL_/d*P* offer a unique possibility to estimate the variations in volume accompanying the LLT (*ΔV*_LL_) using a simple Clausius–Clapeyron equation, , where *ΔH*_LL_ denotes the enthalpy of first-order transition. The calculated values of *ΔV*_LL_ are equal to 0.0029 and 0.0023 cm^3^ g^–1^ for [P_666,14_]Cl and [P_666,14_][Trz], respectively. Interestingly, despite the high value of the d*T*_LL_/d*P* coefficient, the change in volume accompanying LLT is the smallest among all [P_666,14_]^+^ samples tested so far. This is because of the higher *T*_LL_ and markedly smaller transition enthalpies in these materials.

Finally, we reexamine the behavior of the conductivity relaxation times along the LL and LG transitions for [P_666,14_]Cl and [P_666,14_][Trz]. From Figure 5b, it can be seen that both log *τ*_*σ*_(*P*_LL_) and log *τ*_*σ*_(*P*_g_) dependences of equilibrated samples reveal non-isochronal character; i.e., log *τ*_*σ*_ changes as the pressure increases. Specifically, log *τ*_*σ*_(*P*_LL_) decreases linearly, while non-monotonic behavior is observed for log *τ*_*σ*_(*P*_g_). Interestingly, the latter behavior is well-pronounced for the chloride salt and almost negligible for [P_666,14_][Trz]. From this perspective, [P_666,14_]Cl mimics the behavior of other [P_666,14_] ILs with small anions ([SCN]^−^, [DCA]^−^) while almost isochronal behavior of log *τ*_*σ*_(*P*_g_) makes [Trz] salt similar to that of [P_666,14_] ILs with bulky anions ([TCM]^−^ or [TFSI]^−^). One can speculate that pressure makes the diffusion of chloride anions faster (which is visualized as shorter log *τ*_*σ*_(*T*_g_, *P*_g_)) due to the stronger van der Waals interactions between the cation alkyl chains. Therefore, more free space is available for anion motions. However, above the pressure limit of around 360 MPa, the Cl^–^ slows due to the reduced free volume, *V*_free_. At the same time, for [P_666,14_][Trz], the alkyl chain arrangements are more complex, which results in irregular channels for anion transport being insensitive to pressure changes. In this scenario, anions still move faster than cations, making the system decoupled; however, squeezing does not change them much, resulting in log *τ*_*σ*_(*T*_g_, *P*_g_) being almost isochronal.

## Conclusions

In summary, we investigated the liquid–liquid (LL) and liquid–glass (LG) transitions of two phosphonium ionic liquids comprising the same large amphiphilic cation [P_666,14_]^+^ and much smaller anions, chloride and 1,2,4-trazolide [Trz]^−^, at ambient and elevated pressure. Our DSC experiments have shown that both examined ILs undergo LLT when cooled below 205 K, and the liquid 2 state vitrifies at 198.5 ± 0.5 K. Independently of the cooling/heating rate the LLT was the only thermal event detected in DSC thermograms. The LLT was also clearly seen in dielectric measurements as an abrupt increase in conductivity relaxation times *τ*_*σ*_, reflecting the time scale of charge transport. Note that *τ*_*σ*_ at LLT was two decades longer for [P_666,14_]Cl and [P_666,14_][Trz] (log *τ*_*σ*_ = −1.25 and log τ_σ_ = −1) compared to other [P_666,14_]-based ILs. Furthermore, it was insensitive to the sample thermal history. The latter can not be said about the liquid–glass transition. Precisely, the kink of *τ*_*σ*_(*T*^–1^) dependence being a manifestation of *T*_g_ was better pronounced for quenched IL compared to the slowly cooled one. Moreover, *τ*_*σ*_(*T*_g_) was faster than 1000 s—the time scale commonly identified with the freezing of ion mobility. This reflects the decoupling between the charge transport and structural dynamics in [P_666,14_]Cl and [P_666,14_][Trz] originating from the fast diffusion of anions through the channels formed in the cation matrix. Our high-pressure dielectric experiments show that isothermal compression is equally efficient in inducing LLT as isobaric cooling. However, *τ*_*σ*_(*P*_LL_) is much shorter than under ambient pressure conditions. Consequently, [P_666,14_]Cl and [P_666,14_][Trz] represent the first cases where the charge transport is no longer isochronal at LLT. Interestingly, a liquid–glass transition is also observed for different *τ*_*σ*_ under isothermal compression at various *T*. Consequently, non-monotonic behavior of decoupling at elevated pressure has been found for [P_666,14_]Cl. On the other hand, for [P_666,14_][Trz], *τ*_*σ*_(*P*_g_) was almost constant. Finally, we found that pressure plays an essential role in the self-organization of a liquid 2 phase. Specifically, the slowly and rapidly compressed IL reveals different time scales of charge transport. While the former evolves over time, the latter can be considered an equilibrated phase. These results pave the way for a better understanding of self-organization in ILs and hence control the liquid–liquid transition in ion-containing systems.

## Supporting Information Available

NMR characteristics of synthesized [P_666,14_][Trz] ionic liquid (PDF)
