# Supplementary material for: Non-Isochronal Behavior of Charge Transport at Liquid–Liquid and Liquid–Glass Transition in Aprotic Ionic Liquids
Source: J Phys Chem B. 2024 May 14;128(20):5118–26. doi: 10.1021/acs.jpcb.4c00939 (PMC11129292; doi:10.1021/acs.jpcb.4c00939)
Supplement: Supplementary file 1 — jp4c00939_si_001.pdf [file jp4c00939_si_001.pdf]

Supporting Information

Non-Isochronal Behavior of Charge Transport at Liquid-Liquid and Liquid-Glass Transition in Aprotic Ionic Liquids

S. Koymeth[1], B. Yao[1], M. Paluch[1], S. Dai[2,3], N. Mokhtarinori[3], M. Swadzba-Kwasny[4], Z. Wojnarowska[1*]

[1]Institute of Physics, the University of Silesia in Katowice, Silesian Center for Education and Interdisciplinary Research, 75 Pułku Piechoty 1A, 41–500 Chorzów, Poland

[2]Chemical Sciences Division, Oak Ridge National Laboratory, Oak Ridge, TN 37831, USA

[3]Department of Chemistry, Institute for Advanced Materials & Manufacturing, University of Tennessee, Knoxville, TN 37996, USA

[4]The QUILL Research Centre, School of Chemistry and Chemical Engineering, The Queen's University of Belfast, David Keir Building, Stranmillis Rd, BT9 5AG Belfast, NI, UK.

Nuclear-magnetic resonance (NMR) was used to characterize synthesized ionic liquids. Liquid NMR spectra were recorded at ambient temperature on a JEOL 400YH spectrometer (400 MHz) in deuterated chloroform. ^{1}H and ^{13}C NMR chemical shifts were referenced to tetramethyl silane (TMS) at 0 ppm.

^{1}H NMR ($CDCl_3$, 399.7 MHz) δ 8.04 (s, 2H, Triz C3 and C5), 2.20–2.01 (m, 8H, PCH_2), 1.48–1.22 (m, 48H, CH_2), 0.94–0.83 ppm (m, 12H, CH_3); ^{13}C NMR ($CDCl_3$, 100.5) δ 150.09, 31.93, 31.06, 30.84, 30.51, 30.36, 29.69, 29.66, 29.63, 29.53, 29.37, 29.31, 28.96, 22.70, 22.35, 21.84, 21.79, 21.74, 19.13, 19.09, 18.62, 18.60, 14.14, 13.94 ppm.

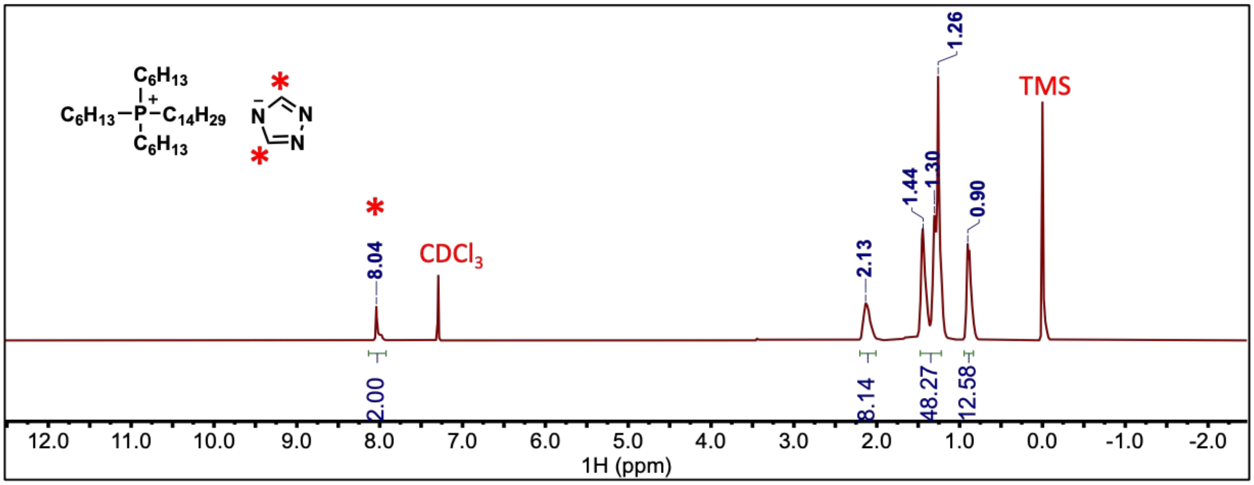

S1: ${}^{1}H$ NMR (399.7 MHz, $CDCl_3$) spectrum of the [P_{66614}] [1,2,4-Triz]).

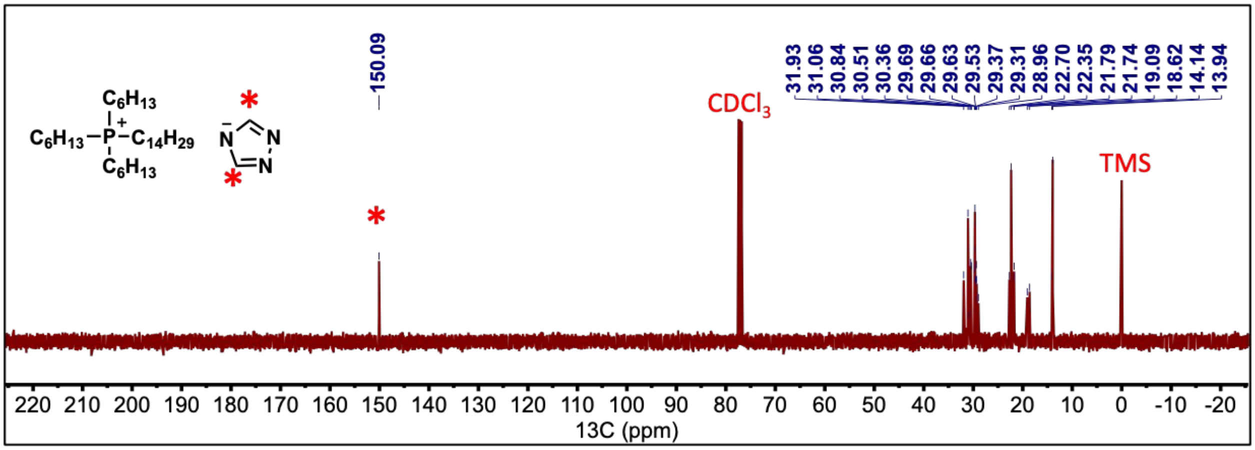

Figure S2: ${}^{13}C$ NMR (100.5 MHz, $CDCl_3$) spectrum of the of the [P_{66614}][1,2,4-Triz]).
